# Violations of Vietnamese laws related to the online marketing of breastmilk substitutes: Detections using a virtual violations detector

**DOI:** 10.1111/mcn.13680

**Published:** 2024-08-28

**Authors:** Kathryn Backholer, Linh Nguyen, Duong Vu, Constance Ching, Phil Baker, Roger Mathisen

**Affiliations:** ^1^ Geelong, Global Centre for Preventive Health and Nutrition, Institute for Health Transformation Deakin University Geelong Victoria Australia; ^2^ Alive & Thrive, Global Nutrition Hanoi Vietnam; ^3^ Alive & Thrive, Global Nutrition Washington District of Columbia USA; ^4^ Institute for Physical Activity and Nutrition, School of Exercise and Nutrition Sciences Deakin University Geelong Victoria Australia

**Keywords:** artificial intelligence, breastfeeding, breastmilk substitutes, code of marketing, commercial milk formula, infant formula, machine learning, policy compliance

## Abstract

Breastfeeding rates in Vietnam, and globally, remain suboptimal. A major contributor to this is the aggressive marketing of commercial milk formulas (CMF), mainly through online media. The Vietnamese Government has implemented legal measures to limit CMF marketing, but these have been difficult to enforce, because of complex online environments. We aimed to quantify the extent and nature of online violations and contradictions in various Vietnamese laws related CMF marketing over 12 months in 2022. Using a cross‐sectional study design, we used an artificial intelligence‐enabled virtual violations detector (VIVID) to monitor official websites and social media pages of 25 breastmilk substitute (BMS) merchandise and distributors, every day for 12 months in 2022. Data were summarised descriptively. We detected more than 3000 online advertisements that violated or contradicted the intent of Vietnamese laws, involving almost 7000 violations of various articles within these laws (average 9.5 violations per day). More than 700 detections were related to CMF products being registered as “supplementary foods” or similar, thereby circumventing Vietnamese CMF marketing laws, because they are not registered as “BMS products. We demonstrate the need to strengthen the design, monitoring and enforcement of existing Vietnamese laws to eliminate mothers” exposure to the exploitative digital marketing of CMF. By turning a highly resource‐intensive task into one that is, automated requiring substantially less resources, our study represents the most comprehensive in Vietnam and internationally on the extent and nature of the online marketing of BMS. VIVID can be applied worldwide to hold industry accountable for the inappropriate marketing of CMF.

## Key messages

- We present the first ever artificial intelligence‐enabled system for improving the implementation of national laws related to the digital marketing of commercial milk formulas (CMF).
- Using Vietnam to apply the system, we detected more than 3000 online advertisements that violated or contradicted the intent of current Vietnamese laws over a 12‐month period.
- Our findings demonstrate the need to strengthen the design, monitoring and enforcement of existing Vietnamese laws to eliminate mothers' exposure to the exploitative digital marketing of CMF.
- These findings should be considered by the Vietnamese Government as they review Advertising and Food Safety Laws.
- The virtual violations detector can be applied worldwide to help governments hold the industry accountable for the inappropriate digital marketing of CMF.

## Graphical abstract

Using an artificial intelligence system virtual violations detector to monitor the digital marketing of commercial milk formulas over 12 months, this study detected more than 3000 breaches to Vietnamese laws. The design and enforcement of current laws for the marketing of commercial milk formulas in Vietnam must be strengthened.

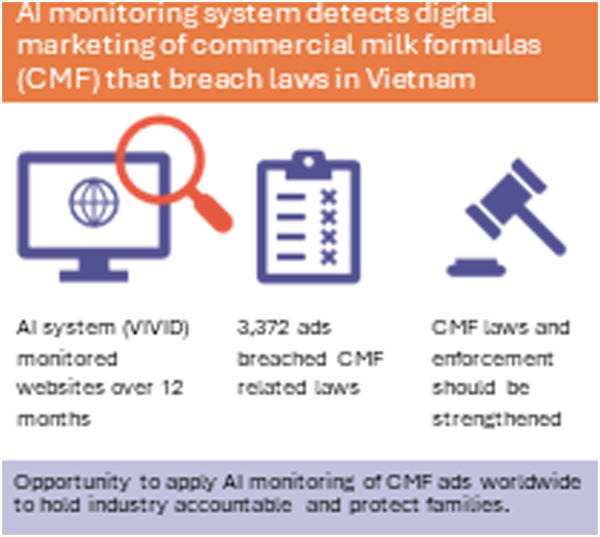

## 1 INTRODUCTION

The World Health Organisation (WHO) recommends that infants initiate breastfeeding at birth, exclusively breastfeed for the first 6 months of life, after which nutritionally adequate and safe complementary foods can be introduced, while breastfeeding continues up to 2 years of age or beyond (World Health Organisation, 2023a). Breastfeeding rates in Vietnam, and globally, are improving but remain far below rates before the marketing of breastmilk substitutes (BMS) greatly intensified in the mid‐20th century. Less than half of infants and young children worldwide meet the WHO recommendations (UNICEF and World Health Organisation, 2022). In contrast, markets of commercial milk formula (CMF), the main form of BMS marketed and consumed worldwide, have proliferated in recent decades, resulting in a US$55 billion global industry, dominated by a few transnational baby food companies, and other companies collaborating and competing at the regional and national levels. These sales are driven by sophisticated highly resourced marketing strategies, including those promoting CMF products as solutions to common infant health and developmental challenges, with little or no supporting evidence (Rollins et al., 2023).

The aggressive marketing of CMF for infants and young children undermines breastfeeding initiation, exclusivity, and duration. In 1981, the World Health Assembly adopted the International Code of Marketing of Breast‐Milk Substitutes (the Code) to ensure safe and adequate infant nutrition by protecting and promoting breastfeeding, protecting bottle or formula fed infants by ensuring proper warnings and instructions on use of products, and ensuring appropriate marketing and distribution of BMS. Since then, the World Health Assembly reviews infant and young feeding progress and makes recommendations accordingly through resolutions every 2 years in response to new evidence and industry marketing practices. Products within the scope of the Code and subsequent relevant resolutions are those targeted to children aged 0–36 months, including CMFs, teats, feeding bottles and commercial complementary foods (CCF) (Guidance, 2017; Rollins et al., 2023). Since the early 1990s, Vietnam has been integrating the Code in various government decrees and laws (WHO et al., 2020). In 2022, these legal measures were reported as being substantially aligned with the Code, but gaps still exist, especially with regard to monitoring and enforcement mechanisms (WHO et al., 2020).

New digital technologies and platforms substantially extend the influence and reach of CMF marketing (Rollins et al., 2023; World Health Organisation, 2022). Online marketing messages are more persuasive and targeted than ever, enabled through mass surveillance of personal data and artificial intelligence (AI) technologies. A recent systematic scoping review of the international literature revealed that CMF products are strategically marketed across various online platforms and channels, in an integrated manner, and this negatively impacts breastfeeding intention and initiation, driving up CMF industry sales (Jones et al., 2022; World Health Organisation, 2022). Meanwhile, current legal measures to regulate CMF marketing through online media are inadequate. According to a scoping review only 24 countries had some regulation of online marketing of CMF outlined in their national Code‐based legal measures (Franco‐Lares et al., 2023). Although Vietnam has relatively comprehensive legal measures in place that prohibit the promotion of BMS, including through online channels, studies show that compliance with these laws is inadequate (WHO et al., 2020). Whilst prior studies are useful to demonstrate noncompliance with existing CMF marketing laws, they are relatively small scale and therefore limited in how much they can conclude on the extent and nature of noncompliance. This is mainly due to the unique logistical challenges related to the highly resource intensive nature of collecting and analysing vast amounts of data in a rapidly changing online environment.

A new approach is needed to help reduce this work burden, and to accelerate and strengthen worldwide monitoring of Code compliance in online environments. Recognising this need, in this study our aim is to use and demonstrate a novel AI enabled virtual violations detector (VIVID), by quantifying the extent and nature of online violations of, or contradictions to various Vietnamese laws related to the marketing of CMF over 12 months in 2022.

## 2 METHODS

### 2.1 Overview

We conducted a cross‐sectional study involving the automated detection of violations or contradictions to the intent of various laws and circulars (through regulatory loopholes) related to the online marketing of CMF between 1 January 2022 and 31 December 2022, using the VIVID tool (FHI Solutions, 2021). We considered a violation or contradiction as (See Table 2 for description of the legal measure and specific violations):

(i) online marketing that violated the Vietnam Code (National Decree 100 and related circulars);
(ii) online marketing of BMS self‐declared as “supplementary” or “functional” foods (meaning that a BMS companies has registered the BMS product as a “supplementary” food under National Decree 15, thereby circumventing the Vietnam Code); and
(iii) online BMS marketing that is in breach of general advertising laws by illegitimately declaring a product as “best,” “the best,” “only,” or “number one” under article 8.11. QC.

According to Decree 38 in 2021 and Decree 117 in 2020, various sanctions for noncompliance to these legal measures are in place (e.g., a violation to National Decree 100 can be subjected to administrative fines, revocation or suspension of advertising permits, publication of violations or legal action), however monitoring and enforcement systems are weak, relying predominantly on civil society complaints (WHO et al., 2020).

### 2.2 Virtual violations detector

VIVID is a virtual assistant that utilises deep‐learning algorithms to detect violations of the International Code and relevant national measures on online platforms, by deploying a combination of object detection, text spotting, multitask classification, and natural language processing (NLP) technologies. For detecting objects in images and videos, VIVID uses a state‐of‐the‐art unified object detection model, YOLO (version 5), which has been trained on a library of relevant images with supervised learning. For identifying text within these images or videos, VIVID uses text spotting (enabled through a PAN++ framework). These tasks are combined using multi‐task classification, using a model trained with MobilenetV2, ResNet, and biLSTM. NLP, enabled through spaCy, allows the analysis of all extracted text to capture the intent of the marketing message. Conditional statements are used to determine if there is a violation and to categorise the violation by the relevant provision in the various legal measures. The system employs a continuous learning process, in which images identified as violating the Vietnam legal measures through VIVID are automatically added to the training data to further enhance algorithm predictive performance. Originally created in Vietnam by Alive & Thrive, an initiative managed by FHI 360 Global Nutrition, and Hekate through a partnership with professionals from Vietnam's Ministry of Health and the Ministry of Information and Communications, VIVID has now been extended with support from the FHI 360 Global Nutrition Innovation Incubator to nine countries, scanning marketing materials on select websites and social media pages that are primarily in English.

### 2.3 Identifying and analysing code violations

VIVID continuously crawls the webpages and fan‐pages (e.g., Facebook) of the top 25 merchandises and distributors of CMF according to market share in Vietnam (identified using Euromonitor International database), which combined, account for 95% of all BMS sales in Vietnam, and 16 online baby product retail platforms. All webpages and fan‐pages represent either local Vietnamese brands or global brands that sell products in Vietnam. Therefore, all advertisements identified on the webpages and fan‐pages target Vietnamese mothers and families. Webpages and fan‐pages were identified using key words with only verified sites.

We extracted all instances identified by VIVID between 1 January 2022 and 31 December 2022 into Microsoft Excel, after which they were verified by one member of the research team (DL) who manually assessed the positive detections and confirmed or rejected the prediction by VIVID. All verified instances were summarised to identify (1) the number of advertisements detected that violated or contradicted the Vietnamese legal measures, according to brand and product type and (2) the provisions of the various laws that were violated. It is of note, that a single advertisement can violate more than one legal measure through violating multiple articles of a given law. For example, a single advertisement may include images and text that promote BMS to the public (article 6.1‐SCT), with a clearly labelled image or drawing of infants and young children, breastfeeding mothers, and feeding bottles (article 8.1.d‐Embe). When assessing violations according to the various articles of the Code, we did not include articles related to healthcare facilities (articles 12.2.b and 12.2.d) as these relate to physical actions within the healthcare facilities that cannot be detected digitally (e.g., accepting BMS samples). The same advertisement could be identified and counted more than once if it was posted more than once by the brand.

## 3 RESULTS

Between 1 January 2022 and 31 December 2022, VIVID scanned 16,511 posts, and detected 3743 online advertisements that were identified in violation or in contradiction to various legal measures related to the marketing of CMF in Vietnam. After manual verification, 3372 advertisements were considered true violations/contradictions (90% true positive rate; Table 1). Most of these were from CMF manufacturers (*n* = 1342), followed by retailers (*n* = 1363), manufacturers of feeding bottles and teats (*n* = 484), and CCF manufacturers (*n* = 183). Companies with the highest number of violations during the year were CMF manufacturers VitaDairy (*n* = 499) and Royal Ausnz (*n* = 335) and online retailers TutiCare (*n* = 573) and Kidsplaza (*n* = 547). Of the top five CMF companies by market share in Vietnam (Vinamilk, Friesland Campina, TH Group, Vinasoy and Nestle), Nestle and Vinamilk were identified as having the most advertisements that violated or contradicted the Vietnam Code.

**Table 1:** Number of advertisements that violated or contradicted the intent of the Vietnam Code, by industry type and brand.

Brand	# of advertisements that violated or contradicted the Vietnamese legal measures
**Commercial milk formula**	
VitaDairy	499
Royal Ausnz	335
Nestlé	73
Blackmores	72
Humana	47
Bellamy's organic	45
Meiji	42
Abbott Grow	39
GuunUp MBP	39
Vinamilk	36
Enfa/Mead Johnson	29
Glico	25
Friso/FrieslandCampina	23
NutiFood	11
Nutricare	7
Goldilac Grow	6
Morinaga	6
Apta	4
Similac/Abbott	3
WAKODO	1
**Sub total**	**1342**
**Bottles and teats**	
YODEE	164
NUK	115
Comotomo	74
Pigeon Vietnam	61
BeBu	46
Chicco Loyalty	14
BABY TATTOO Vietnam	7
Philips Avent	3
**Sub total**	**484**
**Commercial complementary foods**	
GERBER	94
HiPP	87
HEINZ Vietnam	2
**Sub total**	**183**
**Retailer**	
TutiCare	573
Kidsplaza	547
Concung	135
Lazada	45
Soc& Brothers	56
AeonEshop	7
**Sub total**	**1363**
**Total**	**3372**

Because CMF advertisements could violate more than one article across the different legal measures, the 3372 advertisements identified related to 6,810 violations of 19 different articles across the relevant legal measures (Table 2). Of these, the most violated article were those involving information, education, and communications, particularly the use of images, words, or other communications that encourage the use of CMF or bottle feeding or to discourage breastfeeding (4.3.a; *n* = 1052) and information, education and communications with names or logos of CMF, feeding bottles and teats (4.3.c; *n* = 883). Violation through sales promotions, such as free samples, coupons, rewards, gifts, point accumulation for rewards, discounts, or other forms of promotions, were also common (11.2.d; *n* = 1033), as were violations involving the general advertising of CMF for infants and young children under‐24 months (6.1‐SCT; *n* = 995). Advertisements depicting products listed as a “supplementary food” or similar as per ND15 were also common, with 723 instances. There were no violations related to the use of images of a foetus or baby to promote CMF for pregnant women (6.1‐PNMT), nor related to labels on CMF for pregnant women (8.1.d‐PNMT) or on feeding bottles and teats (10.3).

**Table 2:** Number and type of violations of contradictions according to the various articles within the legal measures for CMF marketing in Vietnam.

Article violated or in contradiction of the Vietnam Code and other relevant laws	Corresponding article in violated under the International Code	Violation type	Violation description	Total number of violations
Vietnam Decree 100 and associated circulars				
6.1‐SCT	5.1	Promotion to the public	Advertising of BMS for infants and children under 24 months	995
6.1‐ABS	WHO. Rec 4	2016 Guidance [Foods for Infants and Young Children]	Advertising of CCF for children under‐6 month	217
6.1‐BB	5.1	Promotion to the public	Advertising of feeding bottles and teats	647
6.1‐PNMT	9.1 + 9.2 (b), CMF‐PW	Promotion to the public	Using the image of the foetus or children in the advertisement of CMF for pregnant women	0
8.1.d‐Tuoi	4.2	Labelling	Label of BMS: unclear or absence of information regarding the age group that the product is suitable for	26
8.1.d‐Embe	9.1 + 9.2 (b)	Labelling	Label of BMS: Having images or drawings of infants, breastfeeding mothers, and feeding bottles	343
8.1.d‐Sosanh	9.1 + 9.2 (b)	Labelling	Label of BMS: Use of words or images implying that the product is as good as, or better than, breastmilk in quality or encouraging bottle feeding	144
8.1.d‐PNMT	CMF‐PW	Commercial milk formula for pregnant and lactating women	Label of BMS: Use of images similar to those of labels of CMF for pregnant women	0
9.1.d	WHO. Rec5‐Label	2016 Guidance [Foods for Infants and Young Children]	Label of complementary foods: having no images or drawings of infants, breastfeeding mothers, and feeding bottles; no words or images implying that the product is as good as or better than breastmilk in quality or encouraging bottle feeding;	282
10.3	9.1 + 9.2 (b)	Labelling	Label of feeding bottles and teats: contain images or drawings of newborns and infants, breastfeeding mothers	0
10.3	9.1 + 9.2 (b)	Labelling	Label of feeding bottles and teats: contain images and words implying that these products are similar to the mother's nipples	0
11.2c	5.1	Promotion to the public	Banners, posters, and other leaflets: Display names or logos of breastmilk substitutes on banners, posters and other leaflets in supermarkets, retail stores and medical establishments	0
11.2.d	5.3, 5.4	Promotion to the public	Sales: promotion for BMSs such as giving free samples, coupons, rewards, gifts, point accumulation for rewards, discounts, or any other forms	1033
11.2. dd	7.5 & WHA 49.15	Sponsorships/Conflicts of Interest	Grant scholarships, providing funds for scientific research, training courses, conferences, workshops, music concerts, contests, stage performances, making of films or video clips, telephone counselling services or other forms to disseminate and introduce products, promote the sales or use of BMS	183
4.3.a	4.2	Information, education, and communications	Information, education, and communications (IEC) on nurturing infants: with images, words, or other communications forms to encourage the use of BMS or bottle feeding or discourage breast feeding	1052
6810/356 4.3.b	4.2	Information, education, and communications	IEC: comparisons leading to the conclusion that BMS are as good as or better than breastmilk	93
4.3.c	4.3	Information, education, and communications	IEC: with names or logos of BMS, feeding bottles and teats.	883
Other relevant laws and circulars				
ND15	n/a	Registration of BMS products as a “special” dietary product or a “supplemental” food to circumvent national BMS marketing laws (ND100)	Food for Special Dietary Uses; Food for Special Medical Purposes, Medical Food	723
8.11. QC	Various articles related to health claims	Information, education, and communications; Labelling	Advertising using the words “best”, “the best,” “only,” “number one” or words with similar meaning without legitimate documents proving so as prescribed by the Ministry of Culture, Sports and Tourism.	189
**Total**				**6810**

Nor were there any violations involving names or logos on web‐based banners, posters, and other leaflets in retail stores (their online equivalent; 11.2.c).

## 4 DISCUSSION

Vietnam has a relatively comprehensive legal environment for the marketing of CMF, under the Law on Advertisement, Decree 100, and related legislation. Nevertheless, our results show that these legal measures are inadequate for the online environment. We detected more than 3,000 online advertisements, relating to almost 7000 legal provisions, that either violated or contradicted the intent of the Vietnam Code in the 12 months between January and December 2022. Our study is the most comprehensive to date, in Vietnam and internationally, to report on the extent and nature of the online marketing of CMF and demonstrates the need to strengthen the design, monitoring and enforcement of existing laws to protect Vietnamese mothers from the inappropriate marketing of CMF.

Our findings align with a recent systematic review of the literature on the digital marketing of CMF, which revealed widespread online marketing by CMF companies across the world (Jones et al., 2022). Our study extends these prior studies by including a longer duration of monitoring time and importantly, aligning the marketing content identified with national legal measures. Our findings are not exhaustive, and likely underestimate violations and contradictions, as VIVID only detects marketing messages on major BMS and retailer websites and fan pages, potentially missing smaller BMS suppliers and other forms of BMS marketing. The other forms of marketing that VIVID would not detect include direct marketing messages delivered to caregivers or virtual support groups (e.g., through social media), and use of influencers to promote products and video embedded advertisements. Our findings also underestimate the extent of breaches to the International Code. Although the Vietnamese legal measures prohibiting CMF promotion have been rated as only “substantially” aligned with the International Code, there are some key regulatory gaps when compared to the provisions included in the International Code and relevant WHA resolutions (WHO et al., 2020). For example, the Vietnamese law only includes CMF products for infants and young children up to age 24 months (as opposed to 36 months under the Code) and does not prohibit use of health claims. We did not detect any violations to article 10.3 of the Vietnamese law (corresponding to article 9.1 and 9.2 of the International Code), but we did not report on use of labels depicting images of infants aged 24‐36 months. Future studies could report on violations of both national legal measures and the International Code to identify gaps in existing national legal provisions.

We found that more than 700 BMS products were advertised online as a ‘supplemental food’ (under National Decree 15). This is concerning as doing so precludes a product from being registered as a CMF product, thereby circumventing the Vietnamese law (under National Decree 100 and related circulars). This represents a major legal loophole and lag in enforcement to curb the inappropriate marketing of CMF in Vietnam. Additionally, a CMF product registered as a ‘supplemental food’ does not have to meet national nutritional requirements (as per the national regulation on ‘nutritional formula products’ for infants up to 12 months old ‐ QCVN 11‐1:2012/BYT), placing infants and young children at risk of not receiving the required nutrition.

Our study was enabled through application of VIVID, a world‐first automated virtual violations detector, which turned the highly resource intensive task of monitoring the webpages and fan pages of 25 CMF merchandises and distributors and 12 online retailers, every day, for 12 months, into a lower‐resource task. Whilst human supervision, by way of verifying identified violations from VIVID, is still required, and in fact preferred, to ensure the integrity of the detection process, the time taken to verify positive detections is substantially less compared to a fully manual approach. This obviously translates to significant cost‐savings or more likely makes a practically impossible task practical. VIVID is currently embedded into government monitoring systems in Vietnam, which includes features to help government agencies to track enforcement actions). It has since been expanded to nine other countries, where marketing content is primarily in English, using the International Code as monitoring benchmark.

In countries with Code‐relevant legal measures in place, we recommend adaptation and use of VIVID or similar low‐resource AI‐enabled systems to monitor online media against national laws. With violations detected and enforcement actions tracked, VIVID can also be used to support governments in tracking and imposing sanctions noncompliance, and to identify legal loopholes. In countries without Code‐relevant legal measures in place, VIVID can demonstrate the nature and extent of CMF online marketing, which can be used to advocate for legal measures that are at least aligned with the International Code. The technical requirements Iin technology) to implement VIVID is relatively low after initial set‐up and governments and NGOs can use the system with little to no technological expertise.

It is of note, that the International Code was written and adopted in 1981, before widespread use of the internet, social media, and other online platforms (World Health Organisation, 1981). Whilst subsequent resolutions 54.2 (2001), and the 2016 WHO Guidance (included in Resolution 69.9); Resolution 73.26 (2020), and Resolution 75.21 (2022), all address promotion of CMF and related products on “electronic communications platforms,” the “Internet,” and “digital platforms,” these do not explicitly state *how* the Code should be interpreted for online media. Whilst some countries are introducing measures to interpret or extend existing Code laws to online media (e.g., Viet Nam Decree 70 [Decree, 2021]), these measures do not cover the full extent of online CMF marketing and are not universally applied. In 2022, the 75th World Health Assembly requested that the WHO Director General develop guidance for member states on measures to restrict the online marketing of BMS to ensure that existing and new regulations designed to implement the Code are fit‐for‐purpose in the online world. This guidance was recently published (World Health Organisation, 2023b) and will be critical to support governments around the world to protect against the inappropriate CMF marketing through online media. This should include guidance for holding online platforms accountable for complying with Code laws, as these platforms hold substantial power throughout the CMF marketing chain.

The main strength of our study relates to the novelty of the VIVID tool to automatically detect online CMF advertisements, resulting in a comprehensive list of violations or contradictions to various Vietnamese legal measures over a 12‐month period. Whilst this represents the most comprehensive study to date to monitor potential violations to CMF laws, detection of advertisements are limited to the pre‐specified webpages and fan‐pages. VIVID cannot detect advertisements that are targeted to individuals unless they are also posted by the CMF brands and companies on their own media sites. Nevertheless, we detected a large number of potential violations over the 12‐month monitoring period, demonstrating a major advancement in compliance monitoring for CMF advertising in Vietnam.

Our study shows that CMF companies' use of online marketing practices in Vietnam that violate national legislation is widespread. Such marketing undermines breastfeeding initiation, exclusivity, and duration, placing infants and young children at risk of suboptimal health, growth and development. Low‐resource, automated, monitoring systems such as VIVID hold promise as an important tool for governments around the world as they implement and strengthen CMF laws as they apply in the online environment.

## AUTHOR CONTRIBUTIONS

All authors contributed to the formulation of research questions and study design. Linh Nguyen, Duong Vu and Kathryn Backholer were responsible for data synthesis and analysis. Kathryn Backholer was responsible for writing and drafting the manuscript. All authors provided feedback on the manuscript throughout the drafting process.

## CONFLICT OF INTEREST STATEMENT

The authors declare that there are no conflicts of interest.

## Data Availability

The data that support the findings of this study are available from the corresponding author upon reasonable request.
